# Transformed to myelofibrosis is a risk factor for pulmonary hypertension in Philadelphia chromosome-negative myeloproliferative neoplasms

**DOI:** 10.1007/s00277-025-06334-w

**Published:** 2025-04-21

**Authors:** Dina Suolitiken, Xue Han, Cuicui Feng, Yini Wang

**Affiliations:** Department of Hematology, Capital Medical University Affiliated Beijing Anzhen Hospital, Beijing, 100029 China

**Keywords:** Myelofibrosis, Pulmonary, Hypertension, Myeloproliferative, Neoplasms

## Abstract

Philadelphia chromosome-negative myeloproliferative neoplasms (Ph-MPNs) are a group of malignant clonal disorders originating from bone marrow hematopoietic stem cells, and pulmonary hypertension (PH) is a serious progressive disease often coexisting with Ph-MPNs, with a prevalence ranging from 5 to 50%. The aim of this study was to analyze the prevalence, clinical characteristics, and associated risk factors of PH in 130 patients with Ph-MPNs, to investigate the impact of PH on patients’ prognosis, and to provide a reference for the clinical identification of adverse prognostic factors and early prevention and treatment of PH. One hundred and thirty patients with Ph-MPNs treated at Beijing Anzhen Hospital from January 1, 2020 to December 31, 2024 were included in the study. PH risk was assessed by echocardiography (ECHO), and tricuspid regurgitation velocity (TRV) > 2.8 m/s was used as the criterion for high risk of PH. General information, hematological indices, biochemical indices, gene mutations and echocardiographic data of the patients were collected and statistically analyzed. The overall prevalence of PH among the 130 patients with Ph-MPNs was 15.4%. patients with PMF had the highest prevalence of PH (72.3%), which was significantly higher than that of patients with PV (9.9%) and ET (10.4%) (*P* < 0.05). patients in the PH high-risk group were older, had lower hemoglobin levels, and had a higher prevalence of splenomegaly and secondary myelofibrosis. the PH high-risk group Patients had a significantly higher mortality rate than the normal risk group (20% vs. 1.81%, *P* = 0.0004). Multifactorial Cox regression analysis showed that advanced age (HR = 1.029, *P* = 0.0332) and high risk of PH (HR = 1.034, *P* = 0.0432) were independent risk factors for patient survival.Logistic regression analysis showed that decreased hemoglobin (OR = 0.9657, *P* = 0.0062), splenomegaly (OR = 5.105, *P* = 0.0413) and secondary myelofibrosis (OR = 7.959, *P* = 0.0321) were independent risk factors for high risk of PH. This study revealed the prevalence of PH, risk factors, and their prognostic implications in patients with Ph-MPNs. It is suggested that regular monitoring of changes in relevant risk factors and vigilance and prevention of PH during the treatment of patients with Ph-MPNs are clinically important to improve the prognosis of patients.

## Background

Philadelphia chromosome-negative myeloproliferative neoplasms (Ph- MPNs) are a group of malignant clonal diseases originating from bone marrow hematopoietic stem cells. They mainly include polycythemia vera (PV), essential thrombocythemia (ET), primary myelofibrosis (PMF), chronic myeloid leukemia, chronic neutrophilic leukemia, mastocytosis, chronic eosinophilic leukemia not otherwise specified, and unclassifiable MPNs. Among these, PV, ET, and PMF are classified as the classic Ph- MPNs due to their high degree of similarity. Clinically, they are characterized by the proliferation of one or more peripheral myeloid cell lines, often accompanied by arterial or venous thrombosis, hepatosplenomegaly, and other symptoms [1].

Pulmonary hypertension (PH) is a severe progressive disease that typically increases the risk of heart failure and death [2]. Studies have shown that PH frequently coexists with Ph- MPNs, with an incidence ranging from 5 to 50%. The pathogenesis and influencing factors of PH are considered to be diverse [3–5]. Therefore, in 2022, the European Society of Cardiology and the European Respiratory Society classified PH associated with Ph- MPNs as a category with unclear or multifactorial mechanisms [6]. This study aims to analyze the incidence, clinical characteristics, and related risk factors of PH in 130 Ph- MPN patients, further exploring the impact of PH on patient prognosis. The goal is to provide a reference for identifying poor prognostic factors in clinical practice and for the early prevention and treatment of PH to extend patients’ overall survival.

## Materials and methods

### Case data

Clinical data from 130 patients with Ph-negative MPNs (Ph-MPNs) who were treated at Beijing Anzhen Hospital, Capital Medical University, from January 1, 2020, to December 31, 2024, were collected. Inclusion criteria: patients of any age; diagnosis and classification of Ph-MPNs patients must meet the 2022 WHO criteria [1], primarily confirmed through bone marrow cytomorphology, peripheral blood cell morphology, and bone marrow biopsy; patients who underwent echocardiography during their initial visit or follow-up; relatively complete clinical medical records. Exclusion criteria: secondary polycythemia; secondary thrombocythemia; patients with Philadelphia chromosome-positive status at the time of initial visit; incomplete medical records, no echocardiography performed during the initial visit and follow-up; patients with a history of or concurrent malignancies in other sites.

### Clinical characteristics

The collected patient data included: 1.General information: gender, age, Ph-MPNs subtype, disease duration, history of thrombosis, comorbidities 0.2.Blood tests: white blood cell count, neutrophil count, monocyte count, hemoglobin, hematocrit (HCT), platelet count. 3.Biochemical indicators: lactate dehydrogenase (LDH). 4.Echocardiography: peak tricuspid regurgitation velocity, ejection fraction (EF), tricuspid annular plane systolic excursion (TAPSE, mm), systolic pulmonary artery pressure (sPAP, mmHg). 4.Gene mutations: JAK2 mutation, calreticulin gene (CALR) mutation, thrombopoietin receptor gene (MPL) mutation.

### Determination of pulmonary hypertension

Since almost all Ph-MPNs patients did not undergo hemodynamic diagnosis, the estimated value of systolic pulmonary artery pressure (sPAP) from echocardiography at rest was used, based on the peak tricuspid regurgitation velocity (TRV) and the TRV-derived tricuspid regurgitation pressure gradient (TRPG) — after excluding pulmonary valve stenosis — taking into account the non-invasive estimation of right atrial pressure (RAP), in accordance with the 2022 ESC/ERC guidelines for the diagnosis and treatment of pulmonary hypertension. Among these patients, one patient underwent right heart catheterization for confirmation.

### Statistical analysis

Data were statistically analyzed using SPSS 26.0 software. In univariate analysis, measurement data were tested for normality. Data conforming to a normal distribution were expressed as mean ± standard deviation, and comparisons between groups were performed using independent samples t-tests. Data not conforming to a normal distribution were expressed as medians, and comparisons between groups were performed using the Mann-Whitney U test. Multivariate analysis was conducted using logistic regression analysis. Survival analysis was presented using Kaplan-Meier curves. A P-value < 0.05 was considered statistically significant.

### Incidence of pulmonary hypertension (PH) in Ph-MPNs patients

**Fig. 1:**
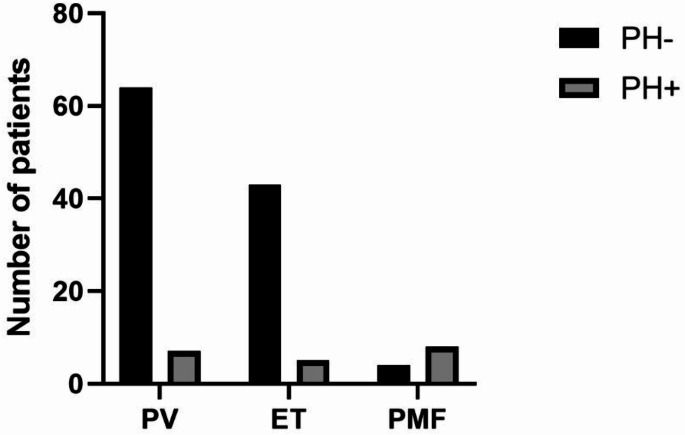
Incidence of Pulmonary Hypertension (PH) in Ph-MPNs Patients

Among the 130 Ph-MPNs patients, classified by disease subtype, there were 71 cases of polycythemia vera (PV), 48 cases of essential thrombocythemia (ET), and 11 cases of primary myelofibrosis (PMF), with PV being the most common subtype. The overall incidence of PH in Ph-MPNs patients was 15.4% (20/130). The incidence of PH in PV, ET, and PMF patients was 9.9% (7/71), 10.4% (5/48), and 72.3% (8/11), respectively. The incidence of PH in PMF patients was significantly higher than in the other two subtypes (all *P* < 0.05) (Fig. 1).

### Demographic characteristics

The cohort consisted of 130 Philadelphia chromosome-negative MPN patients who underwent echocardiography (ECHO) between January 1, 2020, and December 31, 2024. TRV measured by ECHO was used to assess PH risk, with a peak TRV > 2.8 m/s suggesting pulmonary hypertension. Patients were divided into an elevated pulmonary hypertension risk group and a normal pulmonary hypertension risk group. The study included 58 females (44.6%) and 72 males (55.4%). A history of arterial or venous thrombosis was present in 52 patients (40%), and none of the participants were diagnosed with parenchymal lung disease. Due to differences in disease types, treatment regimens for MPN varied slightly between the two groups, but primarily included antiplatelet therapy, anticoagulation, and cytoreductive therapy. The results of the clinical characteristics and epidemiologic data stratified by PH risk are shown in Table 1. Patients with elevated PH risk were older than those with normal PH risk (66 versus 58 years, *P* = 0.038). There were no statistically significant differences between the group with elevated PH risk and the group with normal PH risk with respect to sex, history of thrombosis, white blood cell count, platelet count, JAK2 positivity, or left ventricular ejection fraction (Table 1). During the study period, patients in the elevated PH risk group had a significantly higher mortality rate than patients in the normal PH risk group (20% versus 1.81%, *P* = 0.0004). Among the patients who underwent right heart catheterization (RHC) during the study period, one patient had a mean pulmonary artery pressure of 39.33 mmHg, a pulmonary capillary wedge pressure of 7 mmHg, and a pulmonary vascular resistance of 7.65 Wood units.

**Table 1:** Demographic characteristics

Charateristics	Normal Pulmonary Hypertension Risk Group	Elevated Pulmonary Hypertension Risk Group	t/x^2^	*P*
Age(year)	58.4 ± 14.54	65.65 ± 12.27	2.097	0.038
Sex(Male/Female)	65/45	7/13	3.975	0.0537
WBC(×10^9^)	9.28(1.87–33.39)	10.03(3.47–28.81)	1.556	0.1223
Hemoglobin(g/L)	162(78–237)	134(38–215)	4.047	< 0.0001
Platelet(×10^9^)	448(64-1637)	473(64-1143)	0.2475	0.8049
History of Thrombosis	46(41.8%)	6(30%)	1.227	0.2681
JAK2+	59(53.6%)	8(40%)	1.26	0.2617
Splenomegaly	22(20%)	13(65%)	17.42	< 0.0001
LVEF	64(33–73)	65(45–72)	0.8955	0.3723
TRVmax(m/s)	2.25(1.95–2.8)	3.2(2.9–3.9)	9.462	< 0.0001
sPAP(mmHg)	26(20–38)	53(40–81)	12.94	< 0.0001
TAPSE(mm)	20(10–28)	17.5(10–30)	2.602	0.0111
Transformed to MF	2/106(1.9%)	4/12(33.3%)	22.09	< 0.0001
Antiplatelet Agents and Anticoagulants	80(72.7%)	7(35%)	10.88	0.001
Treatment				
Hydroxyurea	40(36.4%)	7(35%)	0.0136	0.9071
Interferon	23(20.9%)	3(15%)	0.369	0.5434
Ruxolitinib	4(3.6%)	7(35%)	21.49	< 0.0001
Death During Study Period	2(1.81%)	4(20%)	12.71	0.0004

### Main outcome measures

Our primary concern is the prognostic risk associated with PH risk. Given the lack of RHC data in patients with Philadelphia chromosome-negative MPN and the importance of tricuspid regurgitant velocity (TRV) in the most recent European Respiratory Society/European Society of Cardiology guidelines on risk stratification of PH, we used echocardiographic measurements of TRV to assess PH risk. Our primary outcome metric was overall survival (OS), including whether it was an independent risk factor after multifactorial analysis. The secondary outcome metric was the presence or absence of risk factors in patients presenting at high risk for PH, with particular attention to whether thrombotic events and myelofibrosis are risk factors for PH and whether they are indicative of etiologic classification of PH. Finally, we performed exploratory analyses to assess the optimal age for screening for PH and the duration of MPN disease course.

**Fig. 2:**
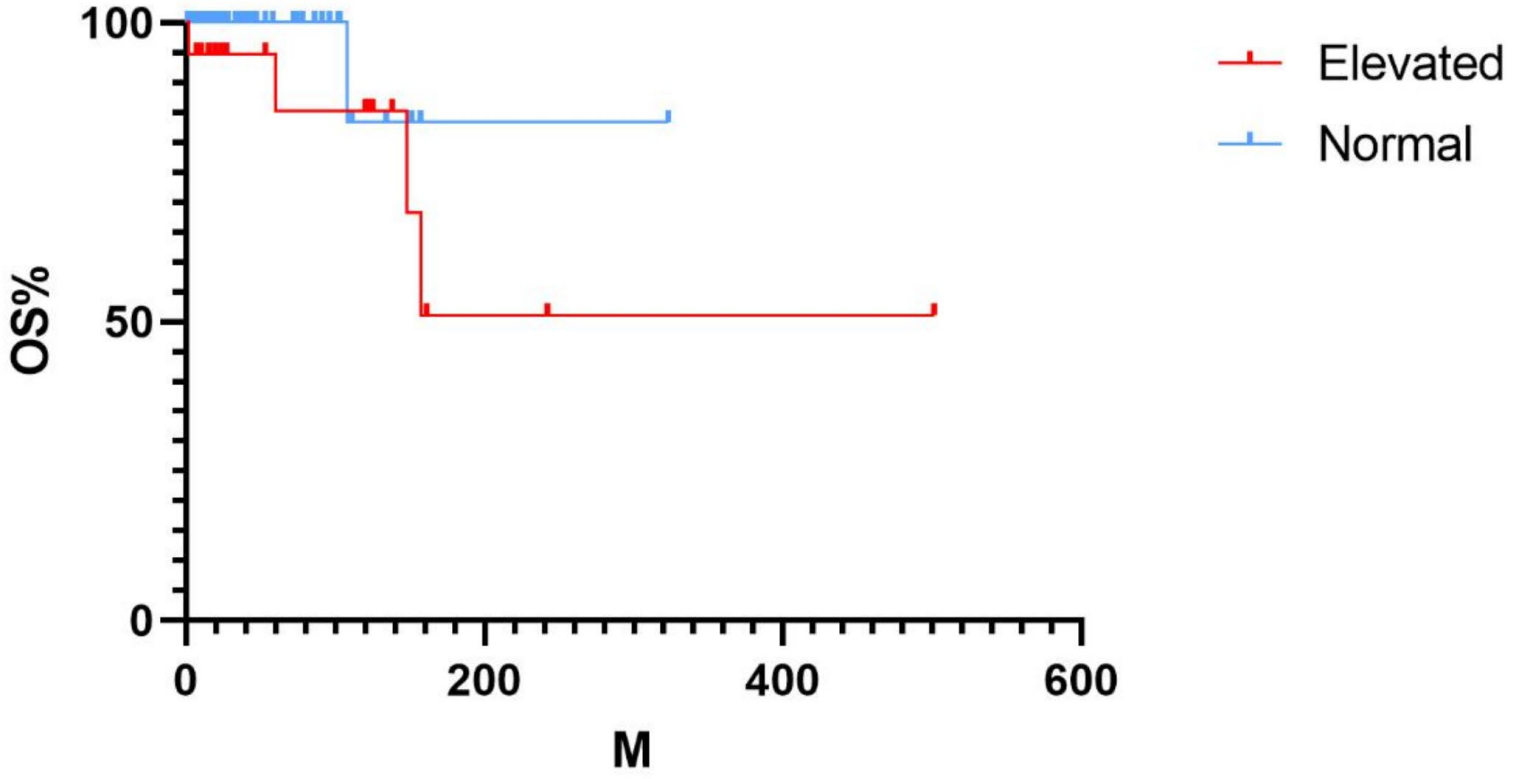
Overall survival in MPN patients with elevated PH risk compared to normal PH risk

Stratifying the Kaplan-Meier survival curves according to the risk of PH (elevated versus normal), patients in the group with elevated PH risk had a higher risk of death than patients in the group with normal PH risk, with a hazard ratio (Mantel-Haenszel) of 4.037 (*P* = 0.0277, Fig. 2). Median survival time was not reached in either group. Cox proportional hazards analysis showed that the elevated PH risk group (assessed by echocardiography) was significantly associated with mortality in univariate analysis (hazard ratio = 4.037 95% confidence interval 0.6173-26.40). This association persisted in a multivariate model that included age, history of thrombosis, and JAK2-positive gene, three standard PH risk variables (hazard ratio = 1.034 95% confidence interval 1.000-1.081) (Table 2). This suggests that echocardiography-based PH risk assessment can independently predict survival in patients with PH.

**Table 2:** Multivariate Cox regression analysis of overall survival in patients with Philadelphia chromosome-negative MPN

Risk factor	Hazard ratio (95% CI)	*P*
Old age	1.029 (1.0118-1.080)	0.0332
Pulmonary hypertension risk	1.034 (1.000-1.081)	0.0432
Thrombosis History	3.516(0.4453–47.31)	0.26
JAK2+	0.5494(0.02186 to 7.817)	0.6526

Exploratory Study.

Multivariate Analysis of Risk Factors for High-Risk Pulmonary Hypertension in Ph-MPN Patients.

By analyzing the clinical data of 130 Philadelphia chromosome-negative MPN patients, divided into high-risk pulmonary hypertension (PH) and normal PH groups, the results showed significant differences in age, hemoglobin levels, splenomegaly, and secondary myelofibrosis indicators between the high-risk PH group and the normal PH group (all *P* < 0.05) (Table 1). Further logistic regression analysis was performed on the factors that showed statistical significance in the univariate analysis. The results revealed that decreased hemoglobin levels, splenomegaly, and secondary myelofibrosis were independent risk factors for the development of high-risk pulmonary hypertension (OR > 1, *P* < 0.05) (Table 3).

**Table 3:** Logistic analysis of patients with Philadelphia chromosome-negative MPN at high risk for pulmonary hypertension

Risk factor	B	SE	χ2	*P*	OR	95%CI
Age	0.008842	0.03274	0.27	0.7871	1.009	0.9459 to 1.079
Hemoglobin	-0.03494	0.01276	2.737	0.0062	0.9657	0.9378 to 0.9880
Splenomegaly	1.63	0.7989	2.041	0.0413	5.105	1.087 to 26.86
TAPSE	-0.02891	0.08655	0.334	0.7384	0.9715	0.8152 to 1.154
Transformed to MF	2.074	0.9679	2.143	0.0321	7.959	1.252 to 60.74

## Discussion

Pulmonary hypertension (PH) is a relatively common complication in patients with Philadelphia chromosome-negative myeloproliferative neoplasms (Ph-MPNs). The occurrence of PH increases the clinical complexity of managing Ph-MPNs, accelerates disease progression and transformation risks, and adversely affects patient prognosis and survival [2]. Studies have shown that the incidence of PH in Ph-MPNs patients is generally high, but there is some variability [5, 7, 8]. This variability is often attributed to factors such as differences in sample size, regional variations, detection methods, and the expertise of the personnel conducting the tests. Subgroup analysis based on Ph-MPNs subtypes revealed that the incidence of PH is highest in primary myelofibrosis (PMF) patients, followed by polycythemia vera (PV) and essential thrombocythemia (ET) patients [3–5]. The differences in disease mechanisms and study populations are the primary reasons for the varying incidence rates.In this study, the overall incidence of PH among the 130 Ph-MPNs patients was 15.4%. Subgroup analysis showed that the incidence of PH in PMF patients (72.3%) was significantly higher than in the other two subtypes, consistent with previous studies.

Our study found that an increased risk of pulmonary hypertension, as detected by echocardiography, is associated with reduced survival rates and shorter survival times in patients with Philadelphia chromosome-negative MPN. Furthermore, a multivariate analysis of factors contributing to poor prognosis in MPN, including age, thrombosis, and JAK2 positivity, revealed that advanced age and a high risk of pulmonary hypertension are independent risk factors. It is well-known that age is a poor prognostic factor in Ph-MPNs patients, and the results were consistent in the enrolled patients of this study [9]. Additionally, the age of patients in the high-risk pulmonary hypertension group was significantly higher than that in the normal-risk group, with statistical significance. Therefore, more aggressive treatment may be necessary for elderly patients with pulmonary hypertension to improve their prognosis. In Ph-MPNs patients, the JAK2-activating somatic mutation is commonly observed, present in over 95% of PV cases and 50–60% of ET and PMF cases [10]. Relevant studies have shown that the JAK2 allele burden is often associated with increased hemoglobin and LDH levels, as well as spleen size in Ph-MPNs patients, factors typically considered risk factors for thrombosis [11]. Additionally, research has found that in MPN model mice, the presence of the JAK2 mutation accelerates the development of PH. Further exploration of the mechanism reveals that this process is due to enhanced activity of neutrophil-derived elastase, which leads to reactive proliferation of pulmonary artery smooth muscle and increased distribution of vascular muscle, resulting in vascular remodeling [12]. However, in this study, the difference in prognosis between JAK2 + and JAK2- patients was not statistically significant, and there was no statistically significant difference in JAK2 + status between the high-risk and normal-risk pulmonary hypertension groups. The formation of microthrombi in pulmonary hypertension and their progression to chronic thromboembolic pulmonary hypertension might initially seem like the most plausible pathophysiological mechanism for its development. Although a history of thrombosis is significant in assessing the prognosis of Ph-MPNs patients, it was not found to be predictive of overall survival in our cohort study, and there was no statistically significant difference in thrombosis history between the high-risk and normal-risk pulmonary hypertension groups [13]. These results suggest that the increased risk of pulmonary hypertension in Ph-MPNs patients is primarily driven by mechanisms other than thrombosis, such as increased blood viscosity and secondary myelofibrosis, and that premature death due to increased pulmonary hypertension risk in Ph-MPNs patients is not caused by thrombotic events or chronic thromboembolic pulmonary hypertension.

The findings of this study indicate that hemoglobin levels, splenomegaly, and secondary myelofibrosis are associated with an increased risk of PH in these patients. These findings suggest that the indicators may be risk factors for the development of PH in these patients.The relationship between the age of patients with hematological disorders and the occurrence of PH has been reported multiple times. Currently, research on the influencing factors of PH in Ph-MPNs patients has yielded diverse results [2, 5].Studies have shown that as patients age, pulmonary artery pressure increases accordingly [5, 14], which in turn leads to the development of PH. In this study, the median age of onset for all patients was 63 years, with the most common age range for PH onset being 65–75 years. This also indicates that older age is a risk factor for the occurrence of PH in these patients. Additionally, a longer disease course may, on one hand, lead to a gradual worsening of the condition, and on the other hand, may also result in an increased incidence of PH. This can exacerbate disease progression and significantly increase the risk of PH in patients with Ph-MPNs [15]. Therefore, special attention should be paid to the development of PH in elderly patients with a long disease course. The development of PH associated with Ph-MPNs may involve multiple pathophysiological mechanisms, including thrombosis, pulmonary vascular remodeling, and extramedullary hematopoiesis. Some studies suggest that higher levels of hematocrit (HCT) can lead to increased blood viscosity, thereby raising the risk of thrombosis. Thrombosis can cause microcirculatory disturbances, which in turn may trigger PH [16] The results of this study indicate that reduced hemoglobin levels are an independent risk factor for high-risk pulmonary hypertension. Therefore, further research is needed to explore the pathophysiological factors contributing to pulmonary hypertension in patients with MPN [12].Splenomegaly usually occurs in patients with MPN combined with portal hypertension, a common complication seen in up to 17% of patients with MPN combined with MF. Patients with MPN/MF with portal hypertension have some unique features compared to other causes of portal hypertension, including splenomegaly without hypersplenism (normal white blood cell and platelet counts), and a hyperdynamic circulation that may be the cause of PVH and PH. Hyperdynamic circulation may be the cause of PVH and PH, which is common in portal hypertension [16, 17]. Although it has been suggested in the literature that MF, especially primary MF, seems to confer the greatest risk of PH, possible mechanisms of PH in patients with myelofibrosis have been investigated [3, 14]. They demonstrated that the development of PH in patients with myelofibrosis may occur after hematopoietic infiltration of the lung parenchyma, thrombocytosis, thromboembolism, left ventricular failure and portal hypertension [18]. Our study proposes that patients who convert to myelofibrosis are also at high risk for developing pulmonary hypertension as a patient-independent risk factor, and therefore both patients with primary myelofibrosis and those who present with conversion to myelofibrosis should be actively screened for pulmonary hypertension.

There are several limitations in our study. Firstly, the relatively small sample size of cohorts limits the statistical power of our analysis. The study involved a limited number of participants, which may reduce the statistical power and generalizability of the findings. Smaller sample sizes can make it harder to detect significant effects or draw definitive conclusions. There may be bias in the selection of study participants that could affect the results of the study, it is possible that the sample in this study is not representative of the broader population, and the results of the study may not apply to all patients with Philadelphia chromosome-negative MPN. The diagnosis of PH was not confirmed using invasive methods, such as right heart catheterization, which is the gold standard for measuring pulmonary artery pressure and diagnosing PH. Non-invasive methods, while useful, may not be as accurate or reliable. Sildenafil was applied to treat pulmonary arterial hypertension in only 1 patient in this study, but the study lacked potential treatments for pulmonary hypertension (PH) in patients with positive Philadelphia chromosomes. This omission leaves questions about the most effective treatment strategies for PH in these patients unanswered. Need for further research: The statement emphasizes the necessity for additional studies to better understand how to screen for and treat PH in patients with Ph-MPNs. This includes defining optimal screening methods to identify PH early and developing evidence-based treatment approaches tailored to this specific patient group.

In conclusion, this study investigated the incidence of pulmonary hypertension, risk factors and the effect of pulmonary hypertension on prognosis in patients with Philadelphia chromosome MPNs, therefore, it is recommended that in the process of actively treating the primary disease in patients with Philadelphia chromosome MPNs, it is necessary to monitor the changes of the relevant risk factors on a regular basis, and to be vigilant for and prevent the occurrence of pulmonary hypertension, which is of certain Clinical significance. In summary, while the study may provide valuable insights into the association between PH and Ph-MPNs, it does not address critical clinical questions about how to manage PH in these patients. Future research is essential to bridge this gap and improve patient outcomes.

## Data Availability

No datasets were generated or analysed during the current study.
